# Editorial: Discrimination of Genuine and Posed Facial Expressions of Emotion

**DOI:** 10.3389/fnins.2021.771529

**Published:** 2021-10-07

**Authors:** Huiyu Zhou, Ling Li, Shiguang Shan, Shuo Wang, Jian K. Liu

**Affiliations:** ^1^School of Computing and Mathematical Sciences, University of Leicester, Leicester, United Kingdom; ^2^School of Computing, University of Kent, Canterbury, United Kingdom; ^3^Institute of Computing Technology, University of Chinese Academy of Sciences, Beijing, China; ^4^Department of Chemical and Biomedical Engineering and Rockefeller Neuroscience Institute, West Virginia University, Morgantown, WV, United States; ^5^School of Computing, University of Leeds, Leeds, United Kingdom

**Keywords:** discrimination, genuine, posed facial expressions, emotion, visual presentation

Facial expressions demonstrate emotional states in interpersonal situations. Evidence shows that part of the facial display reflects the emotional experience that is literally felt by the expresser. Interestingly, human beings are capable of identifying facial expressions of the sensed emotions as a form of intentional deceit to conduct social interaction and to present displays that have the support of others. Staged or posed facial expressions implement an emotion that an expresser intends to convey, where genuine expressions are considered as the companion of spontaneous emotional expressions. The ability to differentiate genuine displays of emotional experience from posed ones is very important for dealing with day-to-day social interactions.

Recent work has been conducted on whether or not people can distinguish between posed and genuine displays of emotion. In spite of few studies to investigate this ability, most prior research suggests that people have the ability to judge genuine and posed facial displays. Unfortunately, previous research has suffered from two major shortcomings: (1) the mixture of staged and genuine displays due to the lack of accounting for possible effects of intentional manipulation, and (2) struggling to consider dynamic aspects when people launch facial stimuli for experimental investigation.

This Research Topic consists of the submission of theoretical and experimental perspectives to broaden understanding of the importance of the discrimination of genuine and posed facial expressions of emotion. Some of them report new theoretical approaches, those from other disciplines of psychology not usually utilized within the discrimination of genuine and staged emotion identification or new theories and designs.

In the article entitled “The role of low-spatial frequency components in the processing of deceptive faces: A study using artificial face models,” Kihara and Takeda investigated how spatial frequency information can be used to interpret true emotion. “A call for the empirical investigation of tear stimuli” authored by Krivan and Thomas presents a study on the necessity for empirical investigations of the differences (or similarities) in response to posed and genuine tearful expressions. Zhang et al. conducted research on “Brain activation in contrasts of micro-expression following emotional contexts” with the prediction that the effect of emotional contexts may be dependent on neural activities. Lander and Butcher's article, entitled “Recognizing genuine from posed facial expressions: Exploring the role of dynamic information and face familiarity,” reported the importance of motion for the recognition of face identity before critically outlining the role of dynamic information in determining facial expression and distinguishing between genuine and posed expression of emotion.

Jia et al. introduced a review of the relevant research including spontaneous vs. posed (SVP) facial expression databases and computer vision based detection methods in their article entitled “Detection of genuine and posed facial expressions of emotion: Databases and methods.” In the article authored by Ron-Angevin et al., “Performance analysis with different types of visual stimuli in a BCI-based speller under an RSVP paradigm,” three different sets of stimuli were assessed under rapid serial visual presentation with the following communication features: white letters, famous faces and neutral pictures. In the article “Identifying emotional expressions: Children's reasoning about pretend emotions of sadness and anger,” Serrat et al. attempted to understand children's capacity of identifying pretend emotions by analyzing different sources of information when interpreting emotions simulated in pretend play contexts. In the research work “Deep neural networks for depression recognition based on 2D and 3D facial expressions under emotional stimulus tasks,” Guo et al. created a large scale dataset with subjects of performing five mood elicitation tasks. They also proposed a novel approach for depression recognition using two different deep belief network models. Finally, this thematic topic includes a survey authored by Webster et al., namely “Review: Posed vs. genuine facial emotion recognition and expression in Autism and implications for intervention,” where the literature in studying the deficits of facial emotion recognition in those with autism spectrum disorder is comprehensively discussed.

The studies presented above have set up a landmark to the research on discrimination of genuine and posed facial expressions of emotion. Moving forward, we anticipate more and more research work on deep and thorough analysis of emotion using emerging artificial intelligence techniques.

## Author Contributions

All authors listed have made a substantial, direct and intellectual contribution to the work, and approved it for publication.

## Conflict of Interest

The authors declare that the research was conducted in the absence of any commercial or financial relationships that could be construed as a potential conflict of interest.

## Publisher's Note

All claims expressed in this article are solely those of the authors and do not necessarily represent those of their affiliated organizations, or those of the publisher, the editors and the reviewers. Any product that may be evaluated in this article, or claim that may be made by its manufacturer, is not guaranteed or endorsed by the publisher.

